# Effect of conflict on atrial fibrillation outcomes in the Middle East: Multicenter international cohort study

**DOI:** 10.1016/j.hroo.2025.06.001

**Published:** 2025-06-10

**Authors:** Ibrahim Antoun, Malik Alta'amreh, Alkassem Alkhayer, Alamer Alkhayer, Aref Jalal Eldin, Georgia R. Layton, Riyaz Somani, G. André Ng, Mustafa Zakkar

**Affiliations:** 1 Department of Cardiovascular Sciences, University of Leicester, Leicester, United Kingdom; 2 Faculty of Medicine, University of Aleppo, Aleppo, Syria; 3 School of Medicine, The University of Jordan, Amman, Jordan; 4 Department of Medicine, University of Tishreen's Hospital, Latakia, Syria; 5 Department of Cardiac Surgery, University Hospitals of Leicester NHS Trust, Glenfield Hospital, Leicester, United Kingdom; 6 Faculty of Medicine, University of Damascus, Damascus, Syria; 7 Department of Cardiology, University Hospitals of Leicester NHS Trust, Glenfield Hospital, Leicester, United Kingdom; 8 Leicester British Heart Foundation Centre of Research Excellence, Leicester, United Kingdom; 9 Leicester National Institute for Health and Care Research Biomedical Research Centre, Leicester, United Kingdom

**Keywords:** Atrial fibrillation, Jordan, Syria, Conflict, Middle East, Outcomes

## Abstract

**Background:**

Atrial fibrillation (AF) outcomes vary depending on the stability of the health care system.

**Objective:**

This study compared AF outcomes in 2 neighboring Levant countries: Syria, a conflict-affected country, and Jordan, a country with a stable health care system.

**Methods:**

We conducted a retrospective observational cohort study of patients with AF from Tishreen University Hospital in Latakia, Syria, and the Jordanian Atrial Fibrillation Registry. Propensity score matching was performed to adjust for baseline characteristics. Primary outcomes included 1-year readmission rates and the 1-month incidence of cerebrovascular accidents (CVAs) or major bleeding.

**Results:**

The study included 2677 patients (657 from Syria and 2020 from Jordan). Syrian patients were younger (median age 60 years vs 70 years; *P* < .001) and had higher rates of smoking (39% vs 14%; *P* < .001) and ischemic heart disease (26% vs 12%; *P* < .001) but lower rates of hypertension and diabetes. One-year readmission rates were significantly higher in Syria (64% vs 9%; *P* < .001), as were the incidences of 1-month CVAs (3% vs 1%; *P* < .001) and major bleeding (4% vs 0.5%; *P* < .001). These differences remained significant after propensity score matching. Compared with Jordanian patients, Syrian patients were associated with a substantial increase in all-cause readmission (odds ratio [OR] 1.8; 95% confidence interval [CI] 1.5–2.6; *P* < .001). Furthermore, Syrian patients had a considerable increase in incidences of 1-month CVA (OR 6.5; 95% CI 2.5–16.5; *P* < .001) and major bleeding (OR 20.6; 95% CI 6.7–63.3; *P* < .001).

**Conclusion:**

AF outcomes are significantly worse in Syria than in Jordan. Health care disruptions contribute to increased readmissions and complications. Strengthening AF management in conflict zones through improved access to medications and structured follow-up is essential to mitigating adverse outcomes.

## Graphical abstract

**Figure:**
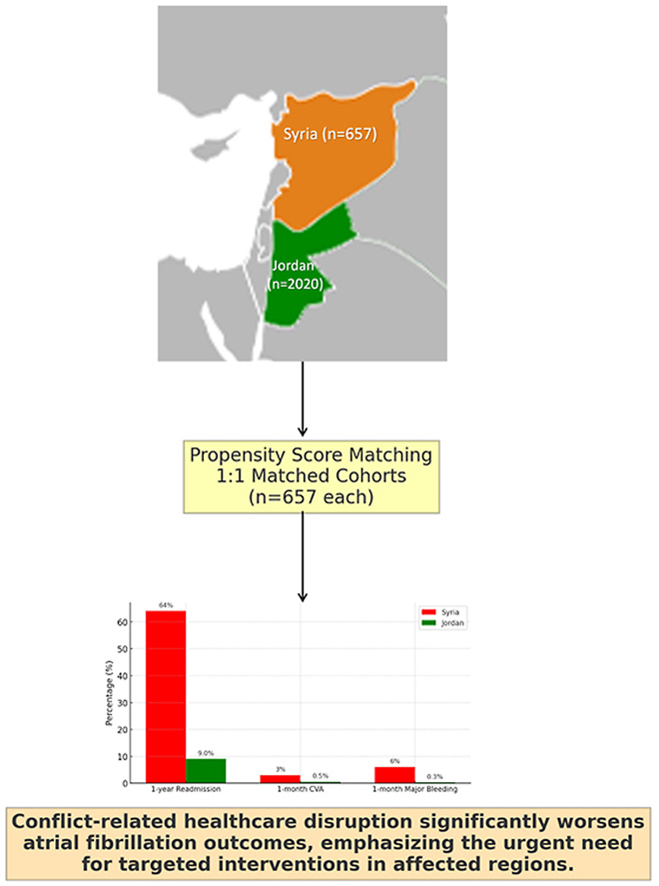

**Key Findings**

▪ Syrian patients had higher rates of readmissions (64% vs 9%), strokes (3% vs 1%), and bleeding (4% vs 0.5%) than did Jordanian patients.
▪ These differences remained significant after propensity score matching, pointing to health care disruptions as the key driver.
▪ Improving access to care and medications is essential to reducing atrial fibrillation–related complications in conflict zones.

## Introduction

Atrial fibrillation (AF) is the most common type of arrhythmia worldwide, and its prevalence in middle- to low-income countries is likely underestimated.^1^ Little data exist on AF management patterns in the Middle East.^2^ Although advances in AF management have improved symptom control, readmission rates continue to increase and are one of the main sources of AF-related financial strain on health care systems.^3^

Syria has been suffering from conflict for the past 14 years. This has left the country deprived of health care funding and resources, which were exacerbated during the coronavirus disease 2019 pandemic and the cholera outbreak.^4^^,^^5^ Less than half of the hospitals operate at full capacity, and more than half of the health care workers are forced to flee the country because of the escalating conflict.^6^ AF management in Syrian hospitals during the ongoing economic and political turmoil is unclear, with a paucity of published inpatient data and outcomes from the Syrian health care system.^7^, ^8^, ^9^, ^10^, ^11^, ^12^, ^13^, ^14^ Furthermore, a recent study demonstrated an AF prevalence of 15% in the emergency department, one of the highest reported rates worldwide.^14^

In contrast, Jordan, a neighboring Levant country that shares long borders with Syria and has a similar cultural and social background, as well as long-standing integration between its citizens, has a relatively stable health care system, with structured registries and data collection efforts to monitor cardiovascular diseases. The Jordanian Atrial Fibrillation (JoFib) Registry provides valuable insights into AF epidemiology, management patterns, and outcomes in Jordanian hospitals. The JoFib study has highlighted key challenges in AF management, including the underuse of anticoagulation therapy and the impact of comorbidities on outcomes.^15^^,^^16^ Although Jordan’s health care system faces financial and infrastructural limitations, it has remained functional and continues to provide specialized cardiovascular care, offering a valuable comparator for assessing the effects of conflict-related health care disruptions in Syria. The impact of prolonged conflict on AF outcomes remains unclear. To address this gap, our study aims to compare the management of AF and patient outcomes between Syria, a conflict-affected country, and Jordan, a neighboring country with a stable health care system. This comparison will provide insight into the consequences of conflict on AF care and may inform strategies to mitigate the adverse effects of health care disruptions in similar settings.

## Methods

### Design and data collection

This is a retrospective, single-center, observational cohort study conducted at Tishreen University Hospital in Latakia, Syria, and nationwide in Jordan. The JoFib study is a prospective, multicenter, observational registry from Jordan that enrolled consecutive patients 18 years or older diagnosed with AF in 18 hospitals and 11 outpatient cardiology clinics from May 25, 2019, through October 25, 2020. Data were collected using a standardized clinical data form at enrollment and at 1, 6, and 12 months after the initial assessment. AF was confirmed by a 12-lead electrocardiogram, a rhythm strip lasting ≥30 seconds, ≥1 episode of AF on ambulatory electrocardiogram monitor, or a past diagnosis by a cardiologist. Patients were followed prospectively for 1 year through outpatient clinic visits, hospital readmissions, or phone calls at 1, 6, and 12 months after enrollment. Data from Syria were collected from Tishreen University Hospital in Latakia between June 2021 and November 2023. The hospital is a large government-operated public institution affiliated with Tishreen University, serving as the primary health care center for the city and surrounding areas. The hospital has 860 beds and provides free health care to Syrian citizens. The hospital cares for ∼50,000–60,000 inpatients annually. The study’s data sources included both paper and electronic hospital records. Medical or cardiology consultants determined the causes of the index admission and readmissions. The research reported in this article adhered to the Declaration of Helsinki. The Syrian part was conducted as part of an audit approved by the hospital board and involved prospective analysis of retrospectively collected anonymized data. Therefore, the ethical committee of Tishreen's University Hospital waived the need for consent (reference 325/A). The Jordanian part of the study was a part of the JoFib registry approved and published previously.^17^ The study was reported according to the Strengthening the Reporting of Observational Studies in Epidemiology (STROBE) statement.^18^

### Outcomes

Our study’s primary outcomes were 1-year readmission rates and their etiology, as well as the 1-month incidence of cerebrovascular accidents (CVAs) or major bleeding, defined according to the Bleeding Academic Research Consortium^19^ criteria. Secondary outcomes included predictors of the primary outcomes.

### Statistical analysis

Continuous variables are expressed as median and interquartile range (IQR), while categorical variables are expressed as count and percentage. The Pearson χ^2^ or Fisher exact test was used to compare categorical variables between groups, and the Student *t* test was used to compare continuous variables. A linear regression model was fitted to estimate the effect of country on readmission at 1 year and CVA and major bleeding at 1 month. Multiple logistic regression models were used to investigate the relationships between 1-year readmission, 1-month CVA, 1-month major bleeding, and various covariates. Propensity score matching between patients from Syria and Jordan was performed using 1:1 nearest-neighbor matching without replacement, based on baseline characteristics from the data set that we determined to be best related to clinical outcomes, which included age, sex, diabetes mellitus, congestive cardiac failure (CCF), current smoking, active thyroid disease, active malignant tumor, chronic kidney disease, and chronic lung disease (Supplemental Figure 1). After matching, all standardized mean differences (SMDs) for covariates were checked using love plots and the adequate balance was set to be below 0.1 (Supplemental Figure 2). After propensity score matching, pre- and postoperative outcomes were compared using the paired Student *t* test or paired Wilcoxon test for continuous variables, the McNemar test for dichotomous variables, and the χ^2^ test for ordinal categorical variables. Variables with <10% missing data were imputed using mean imputation for continuous variables and mode imputation for categorical variables. Outcome variables with missing data were excluded from the relevant analyses. The overall missing data rate for outcome variables was <1.5%.

In addition, a logistic regression model was fitted to investigate the impact of country on outcomes in the matched population. All tests were 2-sided, with an α level of .05 indicating statistical significance. Clinical data were recorded and tabulated using Microsoft Excel (Microsoft Corp, Remond, WA). Statistical analysis was performed using R version 4.4.3 (The R Core Team under the auspices of the R Foundation for Statistical Computing, Vienna, Austria).^20^

## Results

### Baseline characteristics

A total of 2677 patients were included in this study, with 657 (25%) from Syria and 2020 (75%) from Jordan. Baseline characteristics are presented in Table 1. Patients from Syria were significantly younger (median age 60 years; IQR 54–67 years) than those from Jordan (70 years; IQR 60–76 years; *P* < .001). The proportion of male patients was slightly higher in Syria (52% vs 49%; *P* = .001). Comorbidities differed significantly between the 2 cohorts. Syrian patients had higher rates of smoking (39% vs 14%; *P* < .001) and ischemic heart disease (26% vs 12%; *P* < .001) but lower rates of hypertension (34% vs 75%; *P* < .001) and diabetes mellitus (22% vs 44%; *P* < .001). In addition, CCF prevalence was slightly higher in Syria (19% vs 15%; *P* = .04). Syrian patients had significantly lower CHA_2_DS_2_-VASc scores than did their Jordanian counterparts (2 [IQR 1–3] vs 4 [IQR 3–5]^21^; *P* < .001).

**Table 1:** Baseline characteristics of patients with 6-mo readmission vs no readmission after index admission for acute atrial fibrillation

Characteristics	Total (N = 2677)	Syria (n = 657)	Jordan (n = 2020)	*P*	Standardized mean difference
Age (y)	68 (58–75)	60 (54–67)	70 (60–76)	<.001	1.41
Men	1265 (47)	341 (52)	925 (49)	.001	−0.66
Smoking	535 (20)	255 (39)	280 (14)	<.001	0.12
Hypertension	1727 (64)	222 (34)	1506 (75)	<.001	0.51
Ischemic heart disease	408 (15)	171 (26)	237 (12)	<.001	−0.87
Diabetes mellitus	1026 (38)	146 (22)	881 (44)	<.001	0.33
Cerebrovascular disease	447 (17)	123 (19)	324 (16)	.12	−0.52
Congestive heart failure	433 (16)	123 (19)	310 (15)	.04	0.09
Active thyroid disease	224 (8)	23 (3)	202 (10)	<.001	0.09
CHA_2_DS_2_-VASc score	3 (2–4)	2 (1–3)	4 (2–5)	<.001	−0.37
Chronic kidney disease	234 (9)	53 (8)	181 (9)	.5	−1.15
Active malignancy	163 (6)	44 (7)	119 (6)	.4	0.03
Chronic lung disease	153 (6)	72 (11)	82 (4)	<.001	0.22

Values are presented as median (interquartile range) or n (%) unless stated otherwise.

### Primary outcomes for the unmatched data and predictors

In the full cohort, readmission within 1 year, CVA within 1 month, and major bleeding within 1 month occurred in 606 (23%), 38 (1%), and 47 (2%) patients, respectively. When stratified by country, 1-year readmission occurred more frequently in Syrian patients than in Jordanian patients (64% vs 9%; *P* < .001). Similarly, CVA and major bleeding occurred more often in patients from Syria than in those from Jordan (3% vs 1%; *P* < .001 and 4% vs 0.5%; *P* < .001). Multivariable logistic regression models are presented in Table 2. The table shows that patients from Syria had significantly higher odds of adverse outcomes than did those from Jordan. Being from Syria was associated with a 28.7-fold increased risk of 1-year readmission (odds ratio [OR] 28.7; 95% confidence interval [CI] 20.7–28.7; *P* < .001), a 6.5-fold increased risk of CVAs at 1 month (OR 6.5; 95% CI 2.5–16.5; *P* < .001), and a 20.6-fold increased risk of major bleeding at 1 month (OR 20.6; 95% CI 6.7–63.3; *P* < .001). Increasing age was a significant predictor, with each 10-year increase in age correlating with a 1.1-fold higher likelihood of 1-year readmission (OR 1.1; 95% CI 0.9–1.0; *P* < .001) and major bleeding (OR 1.1; 95% CI 1.0–1.1; *P* < .001).

**Table 2:** Logistic regression showing multivariable-adjusted predictors of primary outcomes in the unmatched study cohort

Variable	1-y readmission		1-mo CVA		1-mo major bleeding	
	OR (95% CI)	*P*	OR (95% CI)	*P*	OR (95% CI)	*P*
Syria (vs Jordan)	28.7 (20.7–28.7)	<.001	6.5 (2.5–16.5)	<.001	20.6 (8–56)	<.001
Age (for every 10-y increase)	1 (0.9–1)	<.001	1.1 (1–1.1)	.0	1.1 (1.02–1.2)	<.001
Men (vs women)	0.7 (0.6–0.9)	.02	0.6 (0.3–1.1)	.2	0.7 (0.3–1.5)	.42
Current smoking (yes vs no)	1 (0.8–1.4)	.85	1 (0.4–2.3)	>.99	2.1 (1.01–4.5)	.05
Hypertension (yes vs no)	0.8 (0.5–1.1)	.18	0.8 (0.3–1.9)	.6	1.3 (0.5–2.9)	.57
Ischemic heart disease (yes vs no)	1.4 (1–1.4)	.05	0.7 (0.3–1.9)	.5	1.8 (0.8–4.1)	.14
Congestive cardiac failure (yes vs no)	1.7 (1.2–2.4)	<.001	0.7 (0.2–1.9)	.4	0.8 (0.3–2.1)	.72
Diabetes mellitus (yes vs no)	2.1 (1.5–2.7)	<.001	1.2 (0.6–25)	.6	1.7 (0.9–3.4)	.12
CVA (yes vs no)	0.8 (0.5–1.3)	.45	1.3 (0.4–4.4)	.62	0.7 (0.2–2.6)	.63
Active thyroid disease (yes vs no)	0.8 (0.5–1.3)	.39	1.4 (0.4–4.7)	.61	1 (0.9–1)	.99
CHA_2_DS_2_-VASc score (for every 1-point increase)	1 (0.9–1.1)	.85	0.9 (0.6–1.4)	.70	0.8 (0.5–1.2)	.37
Chronic kidney disease (yes vs no)	1 (0.9–2)	.06	1.4 (0.5–3.9)	.58	0.4 (0.5–3.1)	.39
Active malignancy (yes vs no)	1.7 (1.1–2.6)	.02	2.3 (0.8–6.8)	.13	3.8 (1.2–11.8)	.02
Chronic lung disease (yes vs no)	1.1 (0.7–1.7)	.64	0.8 (0.2–3.7)	.83	1 (0.9–1)	.71

CVA = cerebrovascular accident; OR = odds ratio.

Male sex was associated with a decreased risk of 1-year readmission compared to females (OR 0.7; 95% CI 0.6–0.9; *P* = .02), though it was not a significant predictor of CVAs (*P* = .2) or major bleeding (*P* = .42). Diabetes mellitus independently increased the likelihood of 1-year readmission by 2.1-fold (OR 2.1; 95% CI 1.5–2.7; *P* < .001). CCF was also an independent predictor of 1-year readmission, with a 1.7-fold increased risk (OR 1.7; 95% CI 1.2–2.4; *P* < .001). Active malignancy was associated with a 1.7-fold higher risk of 1-year readmission (OR 1.7; 95% CI 1.1–2.6; *P* = .02) and a 3.8-fold increased risk of major bleeding (OR 3.8; 95% CI 1.2–12.0; *P* = .02).

### Matched population cohort

After matching, 1314 patients (657 per group) were included in the analysis. The patients’ baseline characteristics, the associated SMDs between groups, and propensity scores after matching are reported in Table 3 and Supplemental Figure 1. After matching, there was no residual imbalance in the matching variables—all SMDs were below 0.053.

**Table 3:** Demographic characteristics after propensity score matching

Variable	Syria (n = 657)	Jordan (n = 657)	Standardized mean difference
Age (y)	60.5	60.0	0.52
Men	52%	54%	0.04
Smoking	39%	29%	−0.05
Hypertension	34%	44%	0.20
Ischemic heart disease	26%	21%	−0.22
Diabetes mellitus	22%	19%	0.12
Cerebrovascular disease	19%	16%	0.07
Congestive heart failure	19%	16%	0.06
Active thyroid disease	3%	4%	0.06
CHA_2_DS_2_-VASc score	2.0	2.1	−0.05
Chronic kidney disease	7%	6%	−0.09
Active malignancy	11%	7%	0.03
Chronic lung disease	8%	6%	0.13

### Outcomes for the matched cohort

There were significantly more Syrian patients with all-cause readmission (422 [64%] vs 48 [7%]; *P* < .001), CVA (22 [3%] vs 3 [0.5%]; *P* < .001), and major bleeding (37 [6%] vs 2 [0.3%]; *P* < .001). Compared with Jordanian patients, Syrian patients were associated with a significant increase in all-cause readmission (OR 1.8; 95% CI 1.5–2.6; *P* < .001). Furthermore, Syrian patients had a considerable increase in 1-month CVA (OR 6.5; 95% CI 2.5–16.5; *P* < .001) and major bleeding (OR 20.6; 95% CI 6.7–63.3; *P* < .001).

## Discussion

This study is the first to describe trends in 1-year readmissions, CVAs, and major bleeding in stable and conflict-ridden neighboring countries in West Asia and worldwide. Its main finding is that conflict settings negatively affect AF outcomes, including hospital readmissions, CVAs, and major bleeding.

Our data indicate that Syrian patients had significantly higher 1-year readmission rates than did Jordanian patients, as well as increased incidences of CVAs and major bleeding. Even after propensity score matching, these differences remained significant. This suggests that health care system constraints in Syria, rather than differences in patient baseline characteristics, are key drivers of worse AF outcomes. Prior studies have shown that conflict-related health care disruptions lead to worsening chronic disease outcomes due to reduced access to medications, delays in treatment, and insufficient follow-up care.^22^^,^^23^ Our findings are consistent with this trend, reinforcing the need to strengthen health care systems in conflict zones.

Syria’s worse clinical outcomes can be explained by the ongoing conflict in Syria since 2011, which has primarily affected health infrastructure and led to increased turnover of skilled staff and inadequate numbers of nurses and allied health professionals.^24^ As only half of the primary health care centers and hospitals are fully functional in Syria,^24^ managing risk factors and following up with patients presenting to hospitals with primary AF after discharge is challenging.

In contrast, Jordan has maintained a relatively stable health care system, supported by structured data collection through registries such as JoFib, which facilitates evidence-based AF management.^17^ Jordanian patients had lower readmission rates, likely because of better follow-up care, medication adherence, and greater availability of health care resources. This highlights the critical role of systematic health care approaches in improving AF outcomes, even in resource-limited settings.

Also, access to medications during conflict has been challenging and should be addressed by international health organizations.^25^ Patient engagement in high-risk behaviors, mainly smoking, is correlated with unfavorable AF clinical outcomes.^26^ For example, a recent Syrian study during the conflict demonstrated a smoking rate of 38% in 978 participants who had a mean age of 25 years, which was described as worrying.^27^ Therefore, patient education and addressing these AF risk factors are essential to reducing the readmission burden and optimizing outcomes in resource-depleted communities such as Syria.

Our study underscores the pressing need for both international and local efforts to mitigate the adverse effects of conflict on cardiovascular health. Ensuring medication availability, improving access to follow-up care, and using telemedicine could enhance AF management in Syria and other conflict-affected regions. Furthermore, public health initiatives aimed at tackling modifiable risk factors such as smoking and uncontrolled hypertension could further improve outcomes in resource-limited settings. Strengthening health care training programs to retain skilled professionals and deploying mobile health clinics may also help bridge gaps in AF care. Future research should focus on developing and implementing sustainable models for chronic disease management in these contexts, particularly for cardiovascular diseases, where timely intervention is crucial for reducing morbidity and mortality.

### Limitations

Despite its strengths, our study has several limitations. First, the retrospective design of the Syrian cohort may introduce selection bias, limiting the ability to establish causality between health care system instability and AF outcomes. In addition, differences in data collection methods between the Syrian and Jordanian cohorts could lead to potential inconsistencies in reported outcomes. Although we used propensity score matching to mitigate confounding, individual confounding from unmeasured variables, such as socioeconomic status and health care–seeking behavior, cannot be ruled out. Furthermore, our study was conducted at a single center in Syria, which may not fully represent the broader Syrian population, particularly those in more rural or severely conflict-affected areas. Lastly, follow-up was conducted through hospital visits and phone calls, which may not capture all clinical events, particularly in Syria, where patients might face challenges accessing health care facilities. Future studies should involve multiple centers in Syria and explore alternative strategies for long-term follow-up to enhance the accuracy of outcome reporting. Data on anticoagulation prescription, type, and adherence were not consistently available in the Syrian cohort because of incomplete documentation, limiting our ability to fully explore their role in the observed differences in cerebrovascular and bleeding outcomes. Data on rhythm control strategies (cardioversion, catheter ablation, and antiarrhythmic use) were not available in either cohort. This limits our ability to assess their contribution to clinical outcomes and to quantify procedural disparities between the 2 health care systems.

## Conclusion

This study highlights the significant impact of instability in the health care system on outcomes for AF. Syrian patients had higher readmission rates, CVAs, and major bleeding than did Jordanian patients, likely because of health care disruptions caused by the ongoing conflict. Urgent targeted interventions are needed, including improved access to health care, medication supply, and structured follow-up programs. Addressing modifiable risk factors such as smoking could further enhance outcomes. Future research should investigate sustainable strategies for cardiovascular care in conflict zones.
