# Supplementary material for: Effect of conflict on atrial fibrillation outcomes in the Middle East: Multicenter international cohort study
Source: Heart Rhythm O2. 2025 Jun 10;6(11):1837–42. doi: 10.1016/j.hroo.2025.06.001 (PMC12675000; doi:10.1016/j.hroo.2025.06.001)
Supplement: Supplementary Figures 1 and 2 [file mmc1.docx]

Supplementary Figure S1 visualises the propensity score distribution in the matched treated, matched control, and unmatched control groups.


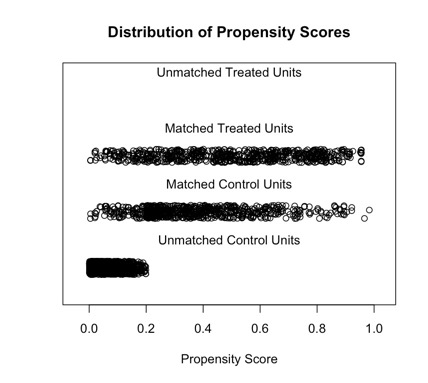


Supplementary Figure S2 visualises the balance in propensity score and all covariates before and after matching. Similar to the results from the table, there are substantial differences in all covariates between smokers and non-smokers before matching, but the difference is close to zero after matching, indicating a very good balance.


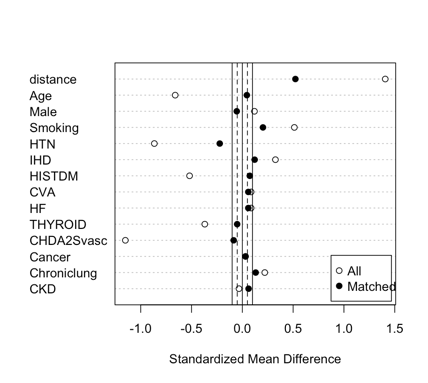


HTN: hypertension. IHD: ischaemic heart disease. HISTDM: diabetes mellitus. CVA: cerebrovascular accident. THYROID: Active thyroid disease. HF: congestive heart failure
